# Easy-to-use clinical tool for survival estimation in Ewing sarcoma at diagnosis and after surgery

**DOI:** 10.1038/s41598-019-46721-8

**Published:** 2019-07-29

**Authors:** S. E. Bosma, C. Lancia, A. J. Rueten-Budde, A. Ranft, H. Gelderblom, M. Fiocco, M. A. J. van de Sande, P. D. S. Dijkstra, U. Dirksen

**Affiliations:** 1 Leiden University Medical Center, department of orthopedics, Leiden, The Netherlands; 2 Leiden University Mathematical Institute, Leiden, The Netherlands; 3 University Hospital Essen, University Duisburg. Essen, Pediatrics III, Sarcoma Centre, West German Cancer Centre, German Cancer Consortium, Essen, Germany; 4 Leiden University Medical Center, department of medical oncology, Leiden, The Netherlands; 5 Leiden University Medical Center, medical statistics/biomedical data sciences, Leiden, The Netherlands; 6 German Cancer Consortium (DKTK), Center Essen, Germany

**Keywords:** Bone cancer, Paediatric cancer, Sarcoma

## Abstract

Accurate survival estimations in Ewing sarcoma are necessary to develop risk- and response adaptive treatment strategies allowing for early decision-making. We aim to develop an easy-to-use survival estimation tool from diagnosis and surgery. A retrospective study of 1314 Ewing sarcoma patients was performed. Associations between prognostic variables at diagnosis/surgery and overall survival (OS), were investigated using Kaplan-Meier and multivariate Cox models. Predictive accuracy was evaluated by cross-validation and Harrell C-statistics. Median follow-up was 7.9 years (95%CI 7.6–8.3). Independent prognostic factors at diagnosis were age, volume, primary tumor localization and disease extent. 5 risk categories (A-E) were identified with 5-year OS of 88% (86–94), 69% (64–74), 57% (50–64), 51% (42–60) and 28% (22–34) respectively. Harrell C-statistic was 0.70. Independent prognostic factors from surgery were age, volume, disease extent and histological response. In categories A-B, 5y OS increased to 92% (87–97) and 79% (71–87) respectively for 100% necrosis and decreased to 76% (67–85) and 62% (55–69) respectively for <100% necrosis. In categories C-E, 5y OS increased to 65% (55–75), 65% (52–78) and 52% (38–66) respectively for ≥90% necrosis and decreased to 38% (22–54), 11% (0–26) and 7% (0–19) respectively for <90% necrosis. We present an easy-to-use survival estimation tool from diagnosis in Ewing sarcoma based on age, volume, primary tumor localization and disease extent. Histological response is a strong additional prognostic factor for OS.

## Introduction

Ewing sarcoma (EwS) is an aggressive bone and soft-tissue tumor predominantly affecting children and young adults^1^. Management rapidly evolved over the last decades, leading to a multimodality approach consisting of chemotherapy, surgery and/or radiotherapy that has become the standard of care. As a result of collaborating trials overall survival (OS) improved drastically, with 10-year OS rates of 55–65% for localized disease. Survival in metastatic disease, present in 20–25% of the patients and usually affecting the lungs (70–80%) and bone/bone marrow (40–45%), is still dismal with 5-year OS varying from 20–35%^2–5^. In primary non-metastatic disease 30–40% of patients experience recurrence, in metastatic disease this number increases to 60–80%. Relapse is mostly systemic (71–73%), followed by combined (12–18%) and local (11–15%) relapse^6,7^. 5-year post-relapse survival is poor, 15–25%, with local recurrence faring better than systemic^6,8,9^.

Personalized medicine encompasses tailoring of treatment based on individual patient characteristics, needs and preferences to improve outcome. Accurate estimations of survival according to the individual patient’s risk profile at different time points are necessary to offer EwS patients the most appropriate treatment, balancing survival and prognosis with toxicity and quality of life. Especially in this young patient population, this balance is essential in our aim to provide the best possible care. Correct survival estimations are difficult and patients and physicians tend to be overoptimistic^10^. Better selection of risk groups and thereby adjusted treatment allows for early decision making, will help improve future outcomes and assists in clinical trial design.

Many studies evaluated the influence of various risk factors on survival in EwS. Only three^8,11,12^ described combining these prognostic factors into risk groups. All three models present shortcomings. They are based on small homogeneous cohorts, that are either not validated or did not include all relevant variables in the model. Keeping these shortcomings in mind, our aim was to develop an easy-to-use survival estimation tool for EwS. Objectives are to: (1) Identify prognostic factors for overall survival from diagnosis and surgery; (2) Develop an accurate baseline prognostic model; (3) Validate the models’ predictive accuracy; (4) Develop a second prognostic model from surgery.

## Methods

This study was reviewed and approved by the Ethical Committee of the Leiden University Medical Center and granted a waiver for informed consent.

### Study population

**Figure 1:**
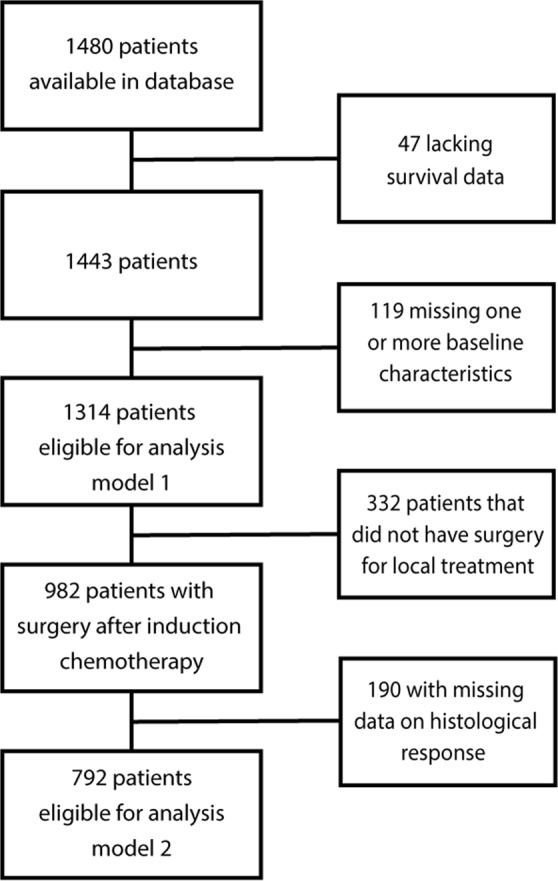
Flowchart inclusion.

A retrospective analysis of patients (randomized and non-randomized) from the EURO-E.W.I.N.G 99 trial database was performed. As detailed in Fig. 1, from 1480 available patients, 166 were excluded due to missing data. Thus, 1314 patients were eligible for analysis at diagnosis. Following induction chemotherapy 982 patients underwent surgery of the primary tumor, 190 were excluded due to missing data, resulting in 792 patients eligible for analysis at surgery. All patients were treated according to the protocol with the aim to administer six cycles of VIDE (vincristine, ifosfamide, doxorubic, etoposide) induction chemotherapy followed by local treatment of the primary tumor. The choice of local treatment, surgery, radiotherapy or both, was left to discretion of the multidisciplinary team. After local treatment patients received maintenance therapy.

### Measures

For accurate risk group stratification large representative and contemporary datasets that closely reflect the target population are needed to enhance the relevance, reproducibility and generalizability of the model^13–17^. Cohorts often contain more variables than can reasonably be used for prediction. Therefore, the most predictive and sensible predictors should be selected. In order to provide all relevant risk factors for such a prognostic model a systematic review^18^ on the current known prognostic factors for overall survival (OS) and event-free survival (EFS) was performed. Based on this systematic review we selected the most predictive and sensible predictors to be included in the univariate analysis. Prognostic factors and outcome were collected prospectively. Patient characteristics included gender and age. Tumor characteristics included location, type, volume at diagnosis, skip lesions, disease extent and number of metastatic lesions. Histological response (percentage necrosis) and resection margins were assessed on the surgical specimen by local pathologists.

### Statistical analysis

The outcome of interest was overall survival (OS) measured from date of diagnosis or date of surgery, until last day of follow-up or date of death. Prognostic factors were evaluated using univariate Cox regression analyses; significant prognostic factors were subsequently included into a multivariate Cox model.

Significant risk factors at diagnosis from the corresponding multivariate Cox model were used to build a stratification scheme of prognostic groups. Prognostic groups were narrowed down into risk categories based on clinical expertise. Another set of risk categories was obtained from the same multivariate Cox model based on predicted survival; a leave-one-out cross-validation framework was used to form cross-validated risk categories on predicted 5-year survival probability^19^. The prognostic value of the clinical risk categories was assessed by comparison with cross-validated risk categories. Details on cross-validation methodology and risk category classification are provided in Supplementary File 1. Correspondence of clinical and cross-validated risk categories was evaluated using precision and recall (Supplementary File 1). Discriminative ability of both stratification schemes was assessed using Harrell’s C-index^20^. Observed survival probabilities of clinical risk categories and corresponding cross-validated counterparts were compared by Kaplan-Meier estimators.

Significant risk factors at surgery from the corresponding univariate analysis were used to build a second multivariate Cox model. Associations were considered significant at a rejection level of 5%. All analyses were performed using SPSS version 23.0, R version 3.4.3, and Python 3.6.5.

## Results

Baseline characteristics and treatment details of the 1314 patients at diagnosis are presented in Table 1. Median follow-up, assessed by reversed Kaplan-Meier method^21^, was 7.9 years (95% confidence interval (CI) 7.6–8.3 years); 531 patients died. Localized disease was present in 916 (69.7%), pulmonary metastasis alone in 182 (13.9%) and extrapulmonary metastasis with or without additional pulmonary metastasis in 216 (16.4%) patients. The 5-year OS was 73% (95%CI, 70–76%), 53% (95%CI, 45–60%) and 28% (95%CI, 22–34%) respectively.

**Table 1:** Patient demographics at diagnosis.

Characteristic	N (%)
**Total**	1314
**Gender**	
Male	792 (60.3)
Female	522 (39.7)
**Age (mean, years + SD)**	16,8 (9.9)
Origin	
Osseous	1107 (84.2)
Extra-osseous	207 (15.8)
Primary tumor localization	
Extremity	499 (38.0)
Upper	108 (8.2)
Lower	391 (29.8)
Axial	815 (62.0)
Pelvic	312 (23.7)
Other	503 (38.3)
Volume at diagnosis	
<200 ml	740 (56.3)
≥200 ml	574 (43.7)
**Skip lesions at diagnosis**	63 (4.8)
Disease extent	
Localized	916 (69.7)
Pulmonary metastasis	182 (13.9)
Extrapulmonary metastasis	216 (16.4)
Number of metastatic lesions	
One	43 (3.3)
≥2	355 (27.0)
Local treatment modality	
Surgery	550 (41.9)
Radiotherapy	193 (14.7)
Surgery + radiotherapy	432 (32.9)
Pre-operative radiotherapy	47 (3.6)
Post-operative radiotherapy	385 (29.3)
Unknown	139 (10.5)

SD = standard deviation.

Continuous variables are presented by means along with corresponding standard deviation between brackets, categorical variables as a number with the percentage between brackets.

### Prognostic factors at diagnosis

Univariate and multivariate Cox proportional hazard models were estimated to investigate the effect of risk factors on OS. Results are shown in Table 2. Univariate analysis showed that age, volume, primary tumor localization, skip lesions, disease extent and number of metastatic lesions are significantly associated with OS. In multivariate analysis age ≥16 years (HR 1.36; 95%CI 1.15–1.62); p < 0.001) volume ≥200 ml (HR 1.50; 95%CI 1.25–1.79; p < 0.001), pelvic location (HR 1.34; 95%CI 1.07–1.67; p = 0.015), pulmonary metastasis only (HR 1.79; 95%CI 1.42–2.27; p < 0.001), extrapulmonary metastasis with or without pulmonary metastasis (HR 3.72; 95%CI 3.02–4.56; p < 0.001) and ≥2 metastatic lesions (HR 2.80; 95%CI 2.33–3.36; p < 0.001) remained significant for OS.

**Table 2:** Hazard ratio (HR) with corresponding 95% confidence interval (CI) from univariate and multivariate analysis at time of diagnosis (n = 1314).

Variables	Univariate analysis		Multivariate analysis	
	HR (95% CI)	p	HR (95% CI)	p
Gender				
Female	1			
Male	1.12 (0.94–1.34)	0.195		
Age				
<16 years	1		1	
≥16 years	1.53 (1.29–1.82)	**<0.001**	1.36 (1.15–1.62)	**<0.001**
Origin				
Osseous	1.13 (0.89–1.45)	0.313		
Extra-osseous	1			
Volume				
<200 ml	1		1	
≥200 ml	1.96 (1.65–2.33)	**<0.001**	1.50 (1.25–1.79)	**<0.001**
Location				
Extremity	1		1	
Axial (excl pelvic)	1.17 (0.95–1.43).	0.148	1.16 (0.94–1.44)	0.178
Pelvic	1.9 (1.54–2.35)	**<0.001**	1.34 (1.07–1.67)	**0.015**
Skiplesions at diagnosis				
No	1		1	
Yes	1.56 (1.10–2.22)	**0.013**	1.11 (0.76–1.60)	0.595
Disease extent				
Localized	1		1	
Pulmonary metastasis	2.05 (1.63–2.58)	**<0.001**	1.79 (1.42–2.27)	**<0.001**
Extrapulmonary metastasis	4.33 (3.56–5.28)	**<0.001**	3.72 (3.02–4.58)	**<0.001**
Number of metastatic lesions				
None	1		1	
One	1.71 (1.1–2.66).	**<0.001**	1.54 (0.98–2.40)	0.059
≥2	3.25 (2.73–3.87)	**<0.001**	2.80 (2.33–3.36)	**<0.001**

### Baseline prognostic model

**Figure 2:**
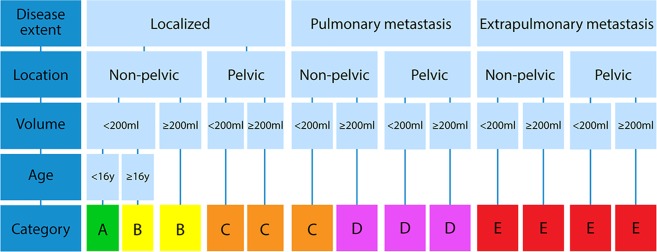
Flowchart for stratification of Ewing sarcoma patients at diagnosis.

Based on the independent prognostic factors at diagnosis (age, volume, location and disease extent), 13 prognostic groups were created and 5 clinically relevant categories (A-E) were estimated. Table 3 provides a detailed description of the prognostic groups and corresponding OS at 3 and 5 years. The 5-year OS for categories A-E was 88% (95%CI 86–94), 69% (95%CI 64–74), 57% (95%CI 50–64), 51% (95%CI 42–60) and 28% (95%CI 22–34) respectively. Figure 2 presents a flowchart to stratify patients at diagnosis. Age only showed strong impact on survival in the first two prognostic groups. In the other prognostic groups survival was similar for patients aged younger than 16 and patients aged 16 and above. Age is therefor only included in the stratification scheme for the first two prognostic groups.

**Table 3:** Overall survival at 3 and 5 years for each prognostic group.

Prognostic group	Disease extent	Location	Volume	Age	N	Overall survival (95%CI)		Category
						3 years	5 years	
1	Localized	Non-pelvic	<200 ml	<16	296	90% (86–94)	88% (84–92)	A
2	Localized	Non-pelvic	<200 ml	≥16	207	80% (75–85)	71% (64–78)	B
3	Localized	Non-pelvic	≥200 ml		243	75% (70–80)	67% (61–73)	B
4	Localized	Pelvic	<200 ml		78	74% (64–84)	62% (50–74)	C
5	Localized	Pelvic	≥200 ml		92	67% (57–77)	53% (43–63)	C
6	Pulmonary	Non-pelvic	<200 ml		57	77% (66–88)	58% (45–71)	C
7	Pulmonary	Non-pelvic	≥200 ml		62	60% (48–72)	48% (36–60)	D
8	Pulmonary	Pelvic	<200 ml		17	82% (67–95)	76% (56–96)	D
9	Pulmonary	Pelvic	≥200 ml		46	54% (39–69)	45% (30–60)	D
10	Extrapulmonary	Non-pelvic	<200 ml		63	36% (24–48)	29% (17–41)	E
11	Extrapulmonary	Non-pelvic	≥200 ml		74	33% (22–44)	31% (20–42)	E
12	Extrapulmonary	Pelvic	<200 ml		22	46% (25–67)	46% (25–67)	E
13	Extrapulmonary	Pelvic	≥200 ml		57	21% (10–32)	17% (7–27)	E

Creation of 13 prognostic groups based on disease extent, tumor localization, volume and age showing overall survival (OS) with corresponding 95% confidence interval (CI) at 3 and 5 years. Last column shows the risk category based on clinical expertise (n = 1314).

**Figure 3:**
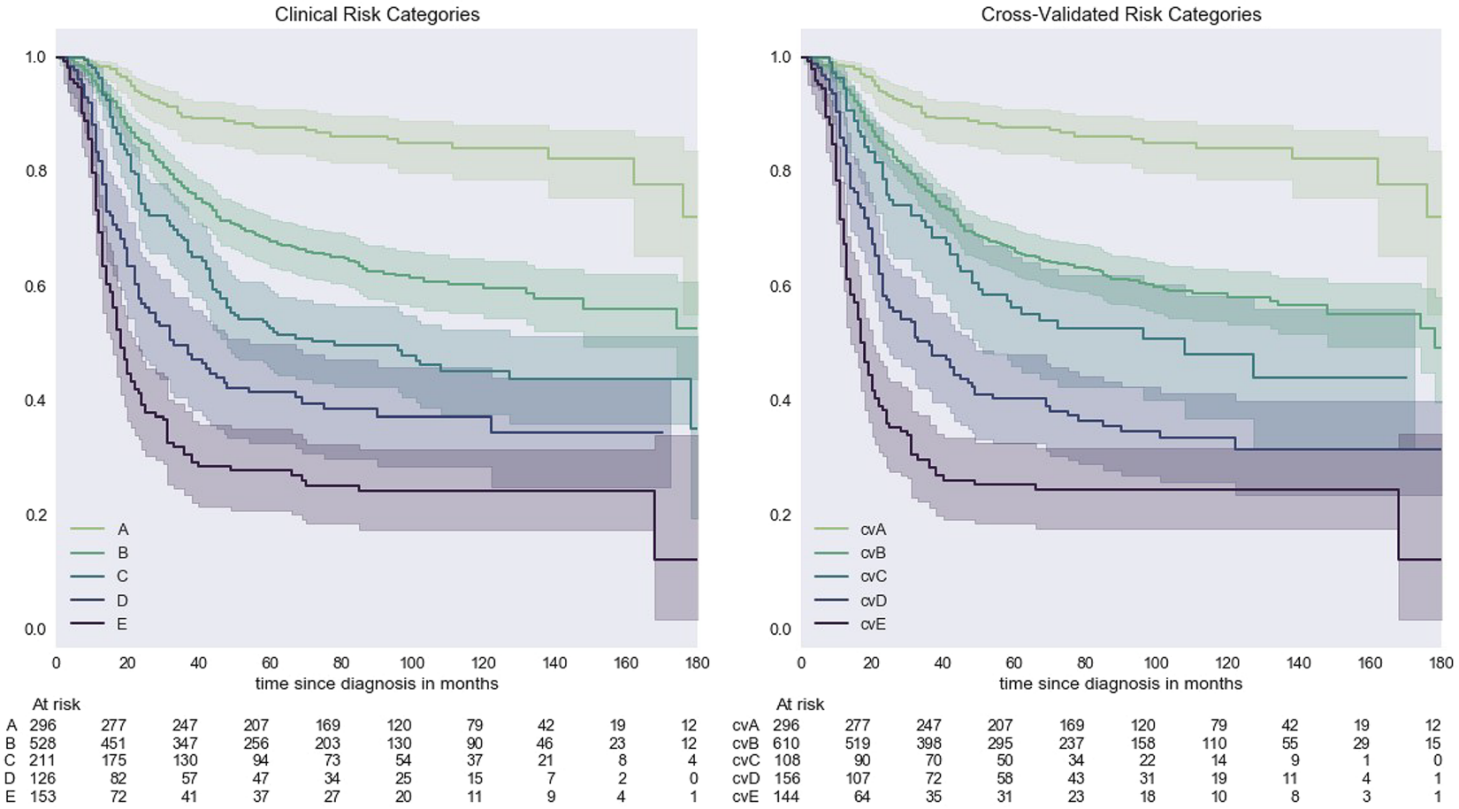
Kaplan-Meier curves for overall survival of clinical risk categories and cross-validated risk categories. Survival is measured in months from diagnosis. On the left the Kaplan-Meier survival curves of the clinical risk categories (**A–E**) based on the 13 prognostic groups. On the right the Kaplan-Meier survival curves of the cross-validated risk categories (cvA-cvE).

Harrell’s C-statistic was 0.70. Discriminatory ability was further evaluated using cross validation. Detailed comparisons of OS in the clinical and cross-validated risk categories at 2, 3 and 5 years are presented in Table 4. Survival probabilities do not show any difference between clinical and cross-validated risk categories. The overall agreement is very good (precision 90.26%; recall 89.57%). Figure 3 illustrates the models’ discrimination ability visualized by the spread of Kaplan-Meier estimates.

**Table 4:** Overall survival at 2, 3 and 5 years for clinical and cross-validated categories.

Category	n	2-year OS (95%CI)		3-year OS (95%CI)		5-year OS (95%CI)	
		Clinical	Cross-validated	Clinical	Cross-validated	Clinical	Cross-validated
A	296	93% (91–96)	93% (91–96)	90% (86–93)	90% (86–83)	88% (84–92)	88% (84–92)
B	450	85% (82–88)	84% (81–87)	77% (73–81)	76% (73–80)	68% (64–72)	66% (62–70)
C	227	74% (68–80)	76% (68–84)	68% (62–75)	70% (62–79)	52% (46–60)	56% (47–67)
D	125	57% (49–66)	57% (50–66)	50% (42–59)	50% (42–58)	41% (33–51)	40% (33–49)
E	216	39% (32–48)	36% (29–45)	30% (24–39)	28% (22–37)	28% (21–36)	25% (19–33)

Detailed comparison of overall survival (OS) with corresponding 95% confidence interval (CI) in each of the clinical and cross-validated risk categories at 2, 3 and 5 years (n = 1314).

### Prognostic factors known at time of surgery

Table 5 shows the effect of prognostic factors known at surgery in univariate and multivariate analysis. Univariate analysis showed that age, volume at diagnosis, primary tumor localization, disease extent, number of metastatic lesions, surgical margin and histological response are significantly associated with OS. In multivariate analysis age ≥16 years (HR 1,38; 95%CI 1,08–1.77; p = 0,01), pulmonary metastasis (HR 1,99; 95%CI 21.47–2,70; p < 0.001), extrapulmonary metastasis with or without pulmonary metastasis (HR 3.18; 95%CI 2.23–4.53; p < 0,001), ≥2 metastatic lesions (HR 2.53; 95%CI 1.93–3.32; p < 0,001) and histological response of 90–99% (HR 1.58; 95%CI 1.16–2.16; p = 0,04) and of <90% (HR 2.90; 95%CI 2,15–3,93; p < 0,001) remained significant prognostic factors for OS.

**Table 5:** Hazard ratio (HR) with corresponding 95% confidence interval (CI) from univariate and multivariate analysis at time of surgery (n = 792).

	Univariate analysis		Multivariate analysis	
	HR (95%CI)	p	HR (95%CI)	p
Gender				
Female	1			
Male	1.08 (0.84–1.37)	0.564		
Age				
<16 years	1		1	
≥16 years	1.53 (1.20–1.94)	<**0.001**	1.38 (1.08–1.77)	**0.010**
Origin				
Osseous	1			
Extra-osseous	1.23 (0.87–1.74)	0.245		
Volume				
<200 ml	1		1	
≥200 ml	1.65 (1.30–2.09)	<**0.001**	1.29 (0.99–1.66)	0.053
Location				
Extremity	1		1	
Axial (excl pelvic)	1.09 (0.82–1.43)	0.564	1.05 (0.79–1.41)	0.735
Pelvic	1.59 (1.18–2.15)	**0.002**	1.30 (0.94–1.79)	0.110
Disease extent				
Localized	1		1	
Pulmonary metastasis	2.09 (1.55–2.81)	<**0.001**	1.99 (1.47–2.70)	<**0.001**
Extrapulmonary metastasis	2.88 (2.03–4.08)	<**0.001**	3.18 (2.23–4.53)	<**0.001**
Number of metastatic lesions				
None	1		1	
One	1.52 (0.85–2.73)	0.159	1.62 (0.90–2.92)	0.108
≥2	2.54 (1.96–3.29)	<**0.001**	2.53 (1.93–3.32)	<**0.001**
Margin status				
Wide	1		1	
Marginal	1.48 (1.08–2.03)	<**0.001**	1.06 (0.76–1.47)	0.736
Intralesional	2.43 (1.55–3.81)	<**0.001**	1.47 (0.91–2.93)	0.120
Histological response				
100%	1		1	
90–99%	1.66 (1.22–2.25)	<**0.001**	1.58 (1.16–2.16)	**0.004**
<90%	2.86 (2.15–3.81)	<**0.001**	2.90 (2.15–3.93)	<**0.001**
Radiotherapy				
No	1			
Pre-operative radiotherapy	1.19 (0.71–1.99)	0.503		
Post-operative radiotherapy	1.10 (0.85–1.41)	0.478		

### Effect of histological response on overall survival

A multivariate Cox model with prognostic factors histological response, risk categories and an interaction term was estimated. The interaction between histological response and risk category was not significant, meaning that the effect of histological response does not vary significantly across the risk categories. The association between histological response and OS was therefore assessed by fitting a Cox model with risk category and histological response, details are presented in Table 6.

**Table 6:** Cox model for overall survival from surgery.

	N	Cox model	p
		HR (95%CI)	
Histological response			
100%	360	1	
90–99%	224	1.57 (1.15–2.12)	**0.004**
<90%	208	3.15 (2.37–4.19)	<**0.001**
Risk category			
A	199	1	
B	316	2.07 (1.42–3.03)	<**0,001**
C	135	3.68 (2.46–5.52)	<**0,001**
D	73	4.38 (2.64–7.28)	<**0,001**
E	69	6.23 (3.72–10.44)	<**0,001**

Hazard ratio (HR) along with 95% confidence interval (CI) (n = 792).

**Figure 4:**
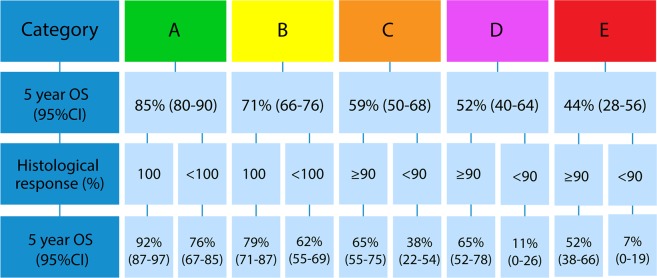
Flowchart for stratification of Ewing sarcoma patients at surgery assessed by Kaplan Meier method.

Figure 4 presents a flowchart to stratify patients at surgery based on the Cox model. For patients in category A with 100% necrosis, 5y OS increased to 92% (95%CI 87–97), but decreased to 76% (95%CI 67–85) when necrosis was <100%. For patients in category B, 5y OS increased to 79% (95%CI 71–87) when necrosis was 100% and decreased to 62% (95%CI 55–69) when necrosis was <100%. In category C, survival increased to 65% (95%CI 55–75) when necrosis was ≥90% and decreased to 38% (95%CI 22–54) when necrosis was <90%. In category D, 5y OS increased to 65% (95%CI 52–78) when necrosis was ≥90% but decreased to 11% (95%CI 0–26) when necrosis was <90%. The same pattern accounts for category E where 5y OS increases to 52% (95%CI 38–66) when necrosis was ≥90% necrosis but drastically decreases to 7% (95%CI 0–19) when necrosis was <90%.

## Discussion

To further improve survival in Ewing sarcoma development of risk- and response adaptive treatment strategies are necessary to allow decision making at different disease stages. Accurate survival estimations are challenging. We developed and validated an easy-to-use survival estimation tool for EwS, based on age, volume, primary tumor localization and disease extent. Furthermore, we show that during the course of treatment survival changes as more information becomes available.

The model presented is based on a cohort of 1314 EwS patients with uniformity in diagnostics and treatment and availability of all relevant prognostic factors. The provided flowcharts are easy-to-use and based on assessable variables. The 13 prognostic groups provide detailed insight in expected survival and could assist in fine-tuning individual treatment. The prognostic groups were narrowed down to 5 risk categories (A-E) based on clinical expertise. The risk categories defined on clinical criteria are consistent with cross-validated risk categories defined on predicted 5-year survival probability. The information gained after surgery offers a second time-point for multidisciplinary decision-making, at this point histological response is an strong additional prognostic factor for OS.

The prognostic significance of the variables in both models has previously been reported. Disease extent is the foundation of the model and strongest prognostic factor in this study. This is consistent with previous studies demonstrating that the presence of metastasis is a strong prognostic factor for survival^22–24^; patients with extrapulmonary metastasis do significantly worse than patients with pulmonary metastasis alone^2,25,26^. Disease extent is also used to define risk groups in previous and current European EwS trials. We also found that primary tumors in the pelvic strongly affect survival, consistent with previously published studies^27^. Other studies suggested an adverse effect on survival for axial localizations (including pelvic) compared to tumours in the extremities^11,28–30^. Volume has also been used to design EwS trails^31^; research shows that larger volumes are associated with poorer survival. Cut-off points at 100 ml^26^ and 150 ml^32^ have been evaluated, but 200 ml seems the most appropriate^33,34^ and was therefore used in this study. Age is an independent prognostic factor for survival in the current study, but only shows strong impact on outcome in two prognostic groups. Cut-of points at 18^22,29,30^ and 14 years^35^ have been evaluated. Strong evidence for a specific cut-off point is lacking. All studies consistently show that older age is associated with poorer survival. We chose 16 years as cut-off, as it is at the interface of pediatric and adult treatment. Histological response, used to tailor treatment in European EwS, is considered of high prognostic value as confirmed in this study. According to literature patients with 100% necrosis have the best survival^28,32^, other studies showed similar results using cut-of points at 95%^36^ and 90% necrosis^33^.

To our knowledge, only three studies described combining prognostic factors into risk groups. Rodriquez-Galindo *et al*.^8^ used Cox proportional hazards models to identify four risk groups in 220 EwS patients based on age (</≥14 years), primary tumor site (pelvic/non-pelvic) and disease extent (localized/isolated lung metastasis/extrapulmonary metastasis). Although based on a small cohort and not validated, our risk groups are similar, with the exception that we added volume to the model. Although they found that tumor size was an independent prognostic factor for survival, they did not include it in the final model. Biswas *et al*.^11^ developed a prognostic model for localized EwS based on 244 patients. Cox models were estimated showing that patients with axial tumors and elevated white blood cell count (WBC) (>11 × 109/L) had poor OS (HR 4.44 (95%CI 2.1–9.4; p < 0.001) and patients with symptoms >4 months, tumor size ≥8 cm and elevated WBC had poor event-free survival (HR 3.89 (95%CI 1.63–9.26; p = 0.002). These models were not validated and are based on a small unmixed cohort limiting its usefulness for clinical decision-making. Additionally, in the systematic review we performed before the start of the current study a consistent association between several biomarkers, such as neutrophil to lymphocyte ratio, hemoglobin and WBC count could not be found, in contrast to the model of Biswas *et al*.^11^ and another study^37^. Lastly Karski *et al*.^12^ derived prognostic groups from 2124 EwS patients in the Surveillance, Epidemiology, and End Results (SEER) database. Using Cox models for OS they constructed five prognostic groups: (1) Localized, <18 years, non-pelvic; (2) Localized, <18 years, pelvic or localized, ≥18 years, White/non-Hispanic; (3) Localized, ≥18 years, other ethnicities; (4) Metastatic, <18 years; (5) Metastatic, ≥18 years. Validation was performed on a cohort of 1680 EwS patients from the Children’s Oncology Group trials, which showed significantly different OS based upon this classification. Although validated, the primary model did not include all relevant variables as the SEER database lacks information on metastatic site. In addition tumor size was missing in 40% of the patients and therefore not included, limiting the strengths of the models.

Limitation of this study include the fact that the local treatment choice was left to discretion of the threatening multidisciplinary teams and might have influenced the results discussed in this article. Secondly, a good prediction model should provide accurate prediction of events by using a comprehensive dataset. In addition, the model should be relatively simple and clinically easy to use. Inaccurate estimates of future events will mislead physicians to provide insufficient treatment. On the other hand, a model with high predictability but which is complex or has too many factors will not be useful. Achieving the optimal balance between predictability and simplicity is the key to a good prediction model^13–17^. Cohorts often contain more variables than can reasonably be used for prediction and for sufficient power one needs at least 10 events per variable. We therefor choose to select the most predictive and sensible predictors to be included in the univariate analysis based on our systematic review^18^. Using a more extensive variable profile could have given useful insights, but we feel that by doing so we would lose simplicity while not improving predictability. Third, surgical margins and histological response were assessed by local pathologists and not by a reference pathologist. Differences between centers in analyzing specimens are possible. Last, the retrospective study design using data form a prospectively performed trial led to some missing data (11%), despite this, a large cohort of EwS patients was available for analysis.

## Conclusion

This study presents an easy-to-use clinical tool to predict OS from diagnosis in EwS, based on age, tumor volume, tumor localization and disease extent. After surgery, the second multidisciplinary decision point, histological response is a strong additional prognostic factor for OS.

## Supplementary information

Supplementary information

## Data Availability

All data generated or analyzed during this study are included in this published article (and its Supplementary Information Files).
