# Supplementary material for: Easy-to-use clinical tool for survival estimation in Ewing sarcoma at diagnosis and after surgery
Source: Sci Rep. 2019 Jul 29;9:11000. doi: 10.1038/s41598-019-46721-8 (PMC6662666; doi:10.1038/s41598-019-46721-8)
Supplement: Supplementary file 1 — Supplementary information [file 41598_2019_46721_MOESM1_ESM.docx]

**Easy-to-use clinical tool for survival estimation in Ewing sarcoma at diagnosis and after surgery****.**

S.E. Bosma, C. Lancia, A.J. Rueten-Budde, A. Ranft, H. Gelderblom, M. Fiocco, M.A.J. van de Sande, S. Dijkstra, U. Dirksen

# **Technical notes on cross-validated risk categories**

Cross-validated risk categories are computed according to the method described in Simon RM, Subramanian J, Li M-C, Menezes S. Using cross-validation to evaluate predictive accuracy of survival risk classifiers based on high-dimensional data. Briefings in Bioinformatics. 2011;12(3), 203-214. This method works with a number of disjoint training/test splitting of the dataset. The original dataset is partitioned in a number of test sets of roughly the same size; these test sets are indicated by T_1_, T_2_, …, T_n_. Test sets have the property that they do not share patients and their union yields the original dataset. For each test set T_i_, the corresponding training set D_i_ is the complement to the original dataset, i.e. the group of patients not contained in the test set. In this manuscript, test sets are composed of just one patient and the corresponding training set is the remaining sample; this is also known as leave-one-out cross-validation.

Given a train/test couple, a Cox model is fitted on the train set and successively used for predicting the 5-year survival probability of patients in the test set (in this manuscript only one patient). The case is then classified in a risk group according to the following scheme:

- A: if predicted 5-year survival is above 80% (included);
- B: if predicted 5-year survival is between 80% (excluded) and 60% (included);
- C: if predicted 5-year survival is between 60% (excluded) and 50% (included);
- D: if predicted 5-year survival is between 50% (excluded) and 30% (included);
- E: if predicted 5-year survival is below 30% (excluded).

The procedure is iterated over each train/test couple of the cross-validation, i.e. for each patient in the sample. After completing this cross-validation, each case will have been classified into one and only one of the five risk groups. Further, each patient will have been assigned a risk category using a model to which they did not contribute to, because they were not in the training set.

The Cox model used in this cross validation is for overall survival on the following four variables: *disease extent* (localized, pulmonary metastases, extrapulmonary metastases), *location* (pelvic, non-pelvic), *volume* (<200ml, ≥200ml), and *age at diagnosis* (<16 years, ≥16 years).
